# Effectiveness of Interventions Promoting Physical Activity and Reducing Sedentary Behavior in Community-Dwelling Older Adults: An Umbrella Review With Application to Saudi Arabia

**DOI:** 10.1007/s44197-023-00111-6

**Published:** 2023-05-18

**Authors:** Saleh A. Alessy, Jesse D. Malkin, Eric A. Finkelstein, Reem AlAhmed, Baian A. Baattaiah, Kelly R. Evenson, Severin Rakic, Volkan Cetinkaya, Christopher H. Herbst, Hazzaa M. Al-Hazzaa, Saleh A. Alqahtani

**Affiliations:** 1 Public Health Department, College of Health Sciences, Saudi Electronic University, Riyadh, Saudi Arabia; 2 Centre for Cancer, Society and Public Health, Faculty of Life Sciences and Medicine, King’s College London, London, UK; 3 The World Bank, Washington, DC USA; 4 Duke-NUS Medical School, Health Services and System Research Program, Singapore, Singapore; 5 King Faisal Specialist Hospital & Research Center, Liver Transplant Center, Riyadh, Saudi Arabia; 6 Department of Physical Therapy, Faculty of Medical Rehabilitation Sciences, King Abdulaziz University, Jeddah, Saudi Arabia; 7 Department of Epidemiology, Gillings School of Global Public Health, University of North Carolina–Chapel Hill, Chapel Hill, North Carolina USA; 8 Lifestyle and Health Research Center, Health Sciences Research Center, Princess Nourah Bint Abdulrahman University, Riyadh, Saudi Arabia; 9 Division of Gastroenterology and Hepatology, Johns Hopkins University, Baltimore, MD USA

**Keywords:** Older population, Physical activity, Physical inactivity, Sedentary behavior, Saudi Arabia, Sitting

## Abstract

**Background:**

As Saudi Arabia is expected to face population aging in the future, the burden of diseases arising from inadequate physical activity (PA) and excess sedentary behavior (SB) may subsequently increase without successful interventions. The present study critically reviews the global literature on the effectiveness of PA interventions targeting community-dwelling older adults to draw on lessons and applications for future interventions in Saudi Arabia.

**Methods:**

This umbrella review of systematic reviews included interventions designed to increase PA and/or reduce SB in community-dwelling older adults. We conducted searches in July 2022 in two electronic databases—PubMed and Embase—and identified relevant peer-reviewed systematic reviews in English.

**Results:**

Fifteen systematic reviews focusing on community-dwelling older adults were included. Several reviews reported that PA- or SB-based interventions, including eHealth interventions (such as automated advice, tele-counseling, digital PA coaching, automated PA tracking and feedback, online resources, online social support, and video demonstrations), mHealth interventions, and non-eHealth interventions (such as goal setting, individualized feedback, motivational sessions, phone calls, face-to-face education, counseling, supervised exercise sessions, sending educational materials to participants’ homes, music, and social marketing programs), were effective in the short term (e.g., ≤ 3 months) but with wide heterogeneity in findings and methodologies. There were limited studies on PA- and SB-based interventions that could be effective for one year or more after the intervention. Most reviews were heavily skewed toward studies carried out in Western communities, limiting their generalizability to Saudi Arabia and other parts of the world.

**Conclusion:**

There is evidence that some PA and SB interventions may be effective in the short term, but high-quality evidence regarding long-term effects is lacking. The cultural, climate, and environmental barriers related to PA and SB in Saudi Arabia require an innovative approach and research to evaluate such interventions in older individuals in the long term.

## Introduction

Due to improvements in health services, living conditions, and work environments, population aging has become a global phenomenon. A growing challenge is to maintain health and fitness throughout the lifespan. There is a large body of evidence showing that regular moderate or vigorous physical activity (PA) can prevent chronic disease [1], slow or reverse sarcopenia [2–5], delay declines in cardiorespiratory fitness [6], reduce the risk of falls [7, 8], and reduce all-cause mortality [9, 10]. Sedentary behavior (SB)—seated or reclining activities that utilize 1.5 or fewer metabolic equivalents [11]—is independently associated with an increased risk of all-cause mortality [12, 13], cardiovascular mortality [12], obesity [14, 15], diabetes mellitus [16, 17], poor cognitive function [18], and poor bone health [19].

Despite the apparent health benefits of engaging in PA and limiting SB, low PA and high SB have been documented in Saudi Arabia [20–22]. Since the number of older citizens is projected to increase dramatically in Saudi Arabia over the next several decades [23], the disease burden due to low PA and high SB will increase without successful interventions. Moreover, several studies on the impact of the COVID-19 pandemic on physical behaviors in Saudi Arabia found that PA further declined due to movement restrictions [24–27].

The World Health Organization recommends that all adults undertake 150–300 min/week of moderate-intensity or 75–150 min/week of vigorous-intensity PA, or some equivalent combination of both and reduce the time spent in SB [28, 29]. Specifically, for older adults, a varied multi-component PA program with strength training and functional balance exercise was strongly recommended [28, 29]. This is similar to recommendations by the Public Health Authority in Saudi Arabia [30] and is aligned with Saudi Vision 2030 objectives for PA and quality of life [31]. Although many initiatives by governmental entities have been implemented to increase PA among all population groups in Saudi Arabia, guidance on what interventions might increase PA and reduce SB among older adults is lacking. The focus of this effort is to review the global literature to draw on lessons and applications for initiatives that could be considered in Saudi Arabia.

## Methods

The search protocol was developed following the Preferred Reporting Items for Systematic Review and Meta-Analysis Protocols (PRISMA-P) statement [32]. We searched the PubMed and Embase databases for systematic reviews of PA, physical inactivity, and SB interventions published since January 1, 2000, with the last day of the search conducted on July 20, 2022. We searched two databases for combinations of the following title words: *physical activity, physical inactivity, sedentary, older, elderly, review, and interventions*.

For inclusion, we required studies to (1) include a systematic review of interventions aimed at increasing PA, reducing physical inactivity, and/or reducing SB among community-dwelling older adults, (2) include eHealth (defined as the use of information and communications technologies in health and health-related fields), mHealth (defined as the use of mobile phones and other wireless technology in health and health-related fields), or any other type of interventions, (3) be published in a peer-reviewed journal, and (4) be published in English. We excluded protocol papers, meeting abstracts, narrative reviews, scoping reviews, reviews that did not focus primarily on intervention effectiveness, reviews that assessed outcomes other than PA or SB, and reviews that targeted small subpopulations of older adults, such as those with cancer or other diseases.

Two reviewers independently screened all the titles and abstracts of studies identified in the database searches. When there was doubt about the inclusion of a study, the full text was retrieved. Thereafter, the same two reviewers assessed the full texts of potentially eligible studies. When there were disagreements, a third reviewer was consulted to finalize the full list of studies to be included in the review.

The following information was abstracted from each review: the type of intervention, the age of the participants, the type(s) of interventions, the number of studies conducted in Western countries, the main results, and the major methodological limitations of the reviewed studies. We did not perform a meta-analysis but described the findings and interpretations of the included studies.

## Results

**Fig. 1:**
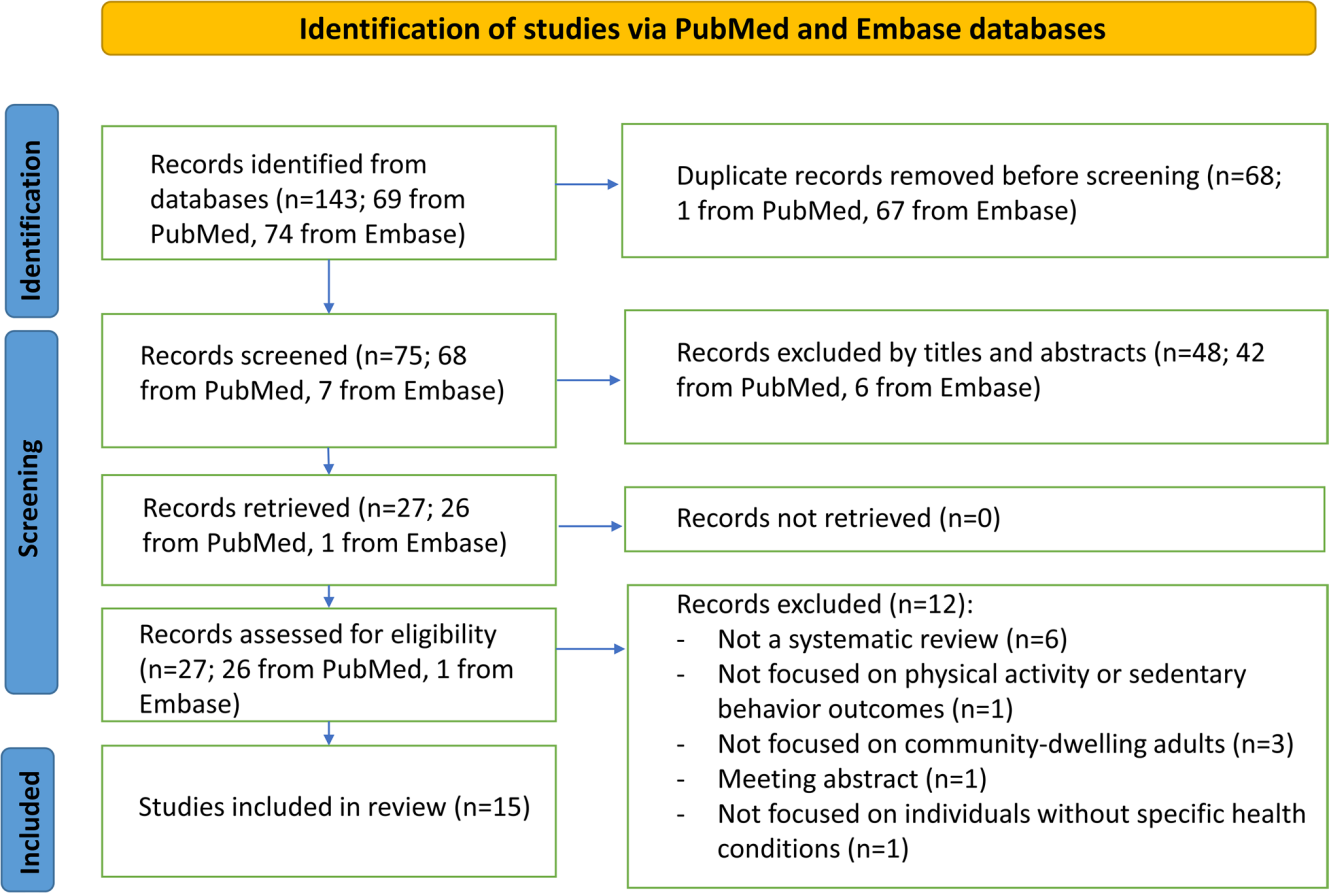
Flowchart of the Literature Search

The initial searches yielded 143 reviews, of which 68 were duplicates. Of the 75 remaining records, we excluded 48 in the title- and abstract-review stage because they were not relevant for the review. We obtained and reviewed the full text of the remaining 27 records. Of these, we excluded 12 studies based on our inclusion/exclusion criteria [33–44]. We included the remaining 15 reviews [45–59]: five reviews of eHealth or mHealth interventions [45–49] and ten reviews of other intervention types [50–59]. Studies were summarized and presented into two main categories: 1) eHealth or mHealth interventions and 2) other interventions. A flowchart of our literature search is shown in Fig. 1, and a summary of the included reviews is provided in Table 1.

**Table 1:** Summary of included reviews

Author, year	Type of intervention	Age of participants (years)	Number of interventions		Main result(s)	Methodological notes
			Included^a^	Conducted in Western countries		
eHealth/mHealth interventions						
Elavsky et al., 2019 [45]	mHealth^b^ (mode: automated messaging, mobile apps, online websites, and their combinations)	≥ 50	52	45	Most studies reported significantly greater PA in the intervention group vs. controls	Almost all studies were ≤ 3 months in duration. 19 studies relied exclusively on self-reported PA. 38 studies comprised populations with specific health conditions, such as cardiovascular disease or obstructive sleep apnea
Kwan et al., 2020 [46]	eHealth^c^ (mode: telecounseling, digital PA recording and tailored advice, video demonstrations and games, online social support, and other)	Mean > 50	38	Unspecified	eHealth interventions are effective at increasing PA	Only three studies tracked outcomes for 12 months or more. About half of the studies relied exclusively on self-reported PA
Muellmann et al., 2018 [47]	eHealth (mode: website, ECA, personal digital assistant, virtual advisor, telephone, text messaging, and other)	≥ 55	20	19	eHealth interventions increased PA in the short run. Evidence on long-term effects is lacking	13 studies relied exclusively on self-reported PA. All but one of the studies were rated as having a moderate or high risk of bias
Stockwell et al., 2019 [48]	eHealth (mode: internet based PA program, personal website and coaching, video games, virtual reality sessions, telephone coaching, ECA, and other)	≥ 50	22	21	eHealth interventions increased PA and moderate-to-vigorous PA and reduced sedentary time	Six studies relied exclusively on self-reported PA. Most studies lasted 16 weeks or less. Among studies that lasted ≥ 6 months, evidence of effectiveness was limited
Yerrakalva et al., 2019 [49]	mHealth (mode: individualized PA prescriptions, mobile and tablet apps, ECA, wearable devices, and their combinations)	≥ 55	6	6	mHealth interventions did not have a statistically significant effect on sedentary time, PA, or fitness	All studies relied on objective measures of outcomes. Few studies were included
Other interventions						
Aunger et al., 2018 [50]	Multiple (mode: motivational sessions, SB education, goals setting, individualized feedback, and phone calls)	Mean ≥ 60	6	5	Device-measured reductions in sitting time were between 3.2% and 5.3% of waking time, or up to 53.9 min per day	The overall quality of the studies was low. The duration of studies ranged from 2 to 8 weeks, precluding conclusions about long-term effects. Three studies relied exclusively on self-reported methods to measure SB
Chase et al., 2020 [51]	Multiple (mode: face-to-face education, counselling sessions, supervised group exercise, nurse consultations, booklets, phone calls and email reminders)	≥ 60	17	16	The SB interventions significantly reduced total sedentary time, but the overall effect was small	Most studies did not meet many of the criteria for internal validity. Eight studies relied solely on self-reported SB. Most studies tracked outcomes for 3 months or less. Only two studies assessed post-intervention outcomes at 6 months or more
Chastin et al., 2021 [52]	Multiple (mode: face-to-face or online education and behaviour change counselling, and self-monitoring)	Mean ≥ 60	7	6	Not clear whether interventions to reduce SB were effective at reducing sedentary time in community-dwelling older adults	High-quality evidence was lacking
Clark et al., 2012 [53]	Music (mode: recorded or live music supporting individual or group exercise sessions)	Mean ≥ 60	12	Unspecified	Evidence did not demonstrate within-session improvements for older adults who listened to music during exercise	Most studies recruited patients from COPD rehabilitation programs or residential care facilities. Rather than measuring PA per day, most studies measured within-session exercise outcomes (e.g., time spent in a single exercise session cycling on a stationary bicycle)
Goethals et al., 2020 [54]	Social marketing (mode: targeted social marketing programs)	> 60	9	7	Several studies reported significant, positive effects of interventions on walking and participation in exercise sessions	Four studies did not directly measure PA or SB. Three studies relied solely on self-reported PA or PA-related outcomes
Grande et al., 2020 [55]	Multiple (mode: general or therapeutic exercise, educational programs, PA coaching or counselling, cognitive behavioural therapies, and feedback)	≥ 60	14	13	Meta-analyses showed that interventions increased PA slightly in the short- and intermediate-term (< 12 months) but not in the long run (≥ 12 months)	Most studies failed to blind examiners and participants. Almost a third of included studies had dropout rates of 15% or higher
Merom et al., 2021 [56]	Workplace (mode: prescribed aerobic programs, one-on-one coaching programs, tai chi or yoga sessions, progressive balance and strength training, and various multicomponent programs)	≥ 50	18	16	Meta-analyses did not show statistically significant differences between the intervention and control groups	Four studies relied on self-reported PA-related outcomes
Müller and Khoo, 2014 [57]	Multiple (mode: print, phone, internet, and other media interventions)	≥ 50	16	16	14 studies reported significant increases in PA	All of the studies relied in whole or part on self-reported PA
Otmanowski and Chase, 2022 [58]	Primary care-based (mode: counselling, goal setting, and education)	Either (a) all participants ≥ 65 or (b) mean age ≥ 70	21	21	Pooled analysis showed a small to moderate positive effect on PA	Only seven of the included studies used objective measures of PA
Sansano-Nadal et al., 2019 [59]	Exercise-based (mode: resistance training, balance training, walking)	Mean ≥ 65	12	Unspecified	Pooled analyses showed that exercise interventions increased PA at six months but not at one- and two-year follow-up assessments	Eight studies measured PA through self-report

^a^In studies with qualitative synthesis and meta-analysis, this column shows the number of interventions included in the qualitative synthesis. Some reviews cited more than one publication that refers to a single intervention

^b^mHealth: the use of mobile phones and other wireless technology in health and health-related fields

^c^eHealth: the use of information and communications technologies in health and health-related fields

*COPD* chronic obstructive pulmonary disease, *ECA* embodied conversational agent, *PA* physical activity, *SB* sedentary behavior.

### eHealth/mHealth Interventions

eHealth is a major focus of the PA and SB literature. Five reviews [45–49] assessed the effects of PA-related eHealth interventions—the interventions administered electronically. A wide range of eHealth interventions was assessed, including automated advice, tele-counseling, digital PA coaching, automated PA tracking and feedback, online resources, online social support, and video demonstrations. Many studies used more than one eHealth strategy at the same time. Some eHealth studies did not promote a specific type of PA, while others specifically aimed to increase walking, stretching, balance, and/or resistance training. Two reviews focused on mHealth interventions, such as automated messaging or use of mobile/tablet apps in combination with wearable devices.

Four of the five eHealth reviews reported similar findings [45–48], with greater PA participation in intervention groups compared to controls. For example, the meta-analysis by Kwan et al. showed that eHealth interventions were associated with a significant increase in PA time compared with the control group results (mean difference = 53.2 min/week, 95% confidence interval [CI] = 30.18–76.21) [46]. This finding is congruent with another meta-analysis that showed increases in moderate-to-vigorous PA equivalent to 52 min/week, which has been shown to have clinical health benefits [48]. A fifth review reported no significant differences between intervention and control groups concerning PA or SB [49].

All four reviews that reported significant intervention effects included a substantial number of studies that relied exclusively on self-reported PA, which correlates poorly with objectively measured PA [60–64]. For example, a meta-analysis of randomized controlled trials by Stockwell et al. [48] showed a positive effect of eHealth interventions on PA but, when trials that relied solely on self-reported PA were excluded, the interventions showed no effect. The one review of eHealth interventions that did not report significant intervention effects excluded studies that relied on self-reported PA or SB [49].

The four eHealth/mHealth reviews that reported significant effects were comprised mainly of studies of ≤ 3 months duration. Short-term increases in PA are unlikely to have long-term health benefits, and evidence suggests that short-term changes in health behaviors are often not sustained. For example, Elavsky et al. noted that the effects of eHealth interventions such as mobile apps, automated messaging, and online resources tended to subside or be reduced in the long term [45]. Among longer-duration studies, high-quality evidence of effectiveness was largely non-existent. Kwan et al. included 38 studies, of which only 3 tracked outcomes for 12 months or more [46]. The review by Stockwell et al. found that many studies were short-term interventions with no follow-up [48]. Muellmann et al. concluded that even though eHealth interventions could effectively promote PA in the short term for adults aged 55 years and above, evidence regarding long-term effects was lacking [47].

In summary, none of the five eHealth reviews cited any high-quality evidence of statistically significant effects of eHealth interventions for one year or beyond. Considering this, Elavsky et al. asked the question of what strategies should be used to encourage long-term use of apps and wearables, given the findings that app use may peak as early as within two weeks of initiation and that one-third of consumers abandon their wearables within 6 months [45].

### Other Interventions

eHealth and mHealth interventions comprise only a portion of interventions focused on PA and SB in community-dwelling older adults. We identified 10 reviews that consisted of other types of interventions, including goal setting, individualized feedback, motivational sessions, phone calls, face-to-face education, counseling, supervised exercise sessions, sending educational materials to participants’ homes, music, and social marketing programs—“the adaptation of commercial marketing technologies to programs designed to influence the voluntary behavior of target audiences to improve their personal welfare and that of society” [54]. Given the heterogeneity of interventions and study populations among these reviews, we present each review separately.

Aunger et al. reviewed various interventions targeting SB in non-working older adults. Interventions included goal setting, individualized feedback, motivational sessions, and phone calls [50]. Although the overall quality of the reviewed studies was deemed poor and half relied solely on self-reported SB, the authors concluded that the interventions could reduce sitting time in non-working older adults by up to 53.9 min per day. Objectively measured reductions in sitting time were between 3.2% and 5.3% of waking time. The duration of studies ranged from 2 to 8 weeks, precluding conclusions about clinical relevance and long-term maintenance of effects.

Chase et al. reviewed a wide range of interventions designed to reduce SB. The interventions included face-to-face education, counseling, and supervised PA [51]. In addition, some interventions entailed sending booklets, DVDs, or other materials to patients’ homes. A meta-analysis showed small but statistically significant reductions in SB among participants in the intervention groups compared to those in the control groups (d =  − 0.25, 95% CI =  − 0.50, 0.00, p = 0.05). However, most studies did not use experimental designs. The studies that used experimental designs did not meet many of the criteria for internal validity, such as blinding of interventionists and assessors. In addition, these studies did not consistently or clearly report strategies to ensure treatment fidelity. Eight of seventeen studies relied on self-reported measures of SB [65–72], and only two had study durations of at least six months [72, 73]. One of the two longer-term studies relied on self-reported SB [72], while the other reported no effect on overall sedentary time [73].

Chastin et al. reviewed a variety of interventions—mainly counseling, goal setting, and education—designed to reduce SB [52]. The authors identified several biases in the reviewed studies. Among the seven studies, only one study blinded participants to allocation to the control group, leading to possible performance and reporting bias. At least two studies did not blind the assessors. Two studies relied solely on self-reported PA. One study did not report on outcomes declared in the methods section, while the prespecified outcomes were only partially reported in two studies. All the studies were relatively small, with sample sizes ranging from 38 to 98 participants. The authors concluded that it was unclear whether interventions to reduce SB were effective. The number of included studies was low, however, and the certainty of the evidence was very low to low, mainly due to inconsistency in findings and imprecision.

The review by Clark et al. had a much narrower focus: the effect of music interventions on PA [53]. The review was motivated by the evidence that listening to music while exercising can increase PA among younger adults, raising the possibility that such an intervention might also be effective among older adults. The narrative synthesis included 12 low- to moderate-quality studies. Overall, three meta-analyses did not demonstrate within-session differences between music and no-music interventions. ‘Within-session’ outcomes—the focus of 10 of the 12 reviewed studies—referred to the amount of exercise that occurred during a brief exercise session, such as the duration of stationary cycling during a cycling session or the number of steps taken during an indoor walking session. These outcomes might have little relation to overall SB or PA, as measured by the number of minutes of moderate-to-vigorous PA per day. Moreover, all but two of the reviewed studies recruited participants from residential care facilities, chronic obstructive pulmonary disease rehabilitation programs, or inpatient rehabilitation programs. The results of such studies may not be generalizable to the broader population of community-dwelling older adults.

Goethals et al. systematically reviewed social marketing interventions to promote PA and PA-related outcomes among adults aged 60 years and above [54]. Nine interventions were included in the analysis. Three relied solely on self-reported PA or PA-related outcomes [74–76], and one of these [76] did not compare people who received the intervention to a control group. Four measured neither PA nor SB [77–80]. One reported no significant differences across communities over 24 months for moderate-to-vigorous PA, even though the number of people attending walking sessions increased in the intervention communities [81]. Only one study—Varma et al. [82]—provided strong evidence of intervention effectiveness. This three-year study found that a community volunteering program in Baltimore (Maryland, US) increased walking activity among older female—but not male—volunteers by 1,500.3 steps per day (95% CI = 77.6, 2,922.9), which was roughly 0.75 miles in distance. According to the authors, a sample size of 702 people was fairly large, and the risk of bias was low.

Grande et al. reviewed a broad array of PA-based interventions, including general or therapeutic exercise, educational programs, PA coaching or counseling, cognitive behavioral therapies, and feedback using objective PA measures such as electronic devices, such as Fitbit [55]. Unlike most other reviews on this topic, the authors limited their analysis to randomized controlled trials that objectively measured PA, mainly through accelerometers and pedometers. Fourteen published trials were included in their analysis. Pooled estimates showed that PA-based interventions were slightly effective compared with no intervention or minimal intervention in the short term (n = 1605; standard mean difference [SMD] = 0.30; 95% CI = 0.17 to 0.43) and intermediate-term (*n* = 895; SMD = 0.27; 95% CI = 0.06 to 0.49). However, there were no statistically significant differences between the intervention and control groups at one year and beyond (n = 323; SMD = 0.19; 95% CI = − 0.03 to 0.41).

Merom et al. reviewed workplace PA interventions that targeted older employees [56]. The interventions involved aerobic activity, strength, balance, and/or flexibility. The results of their meta-analyses showed no significant differences between the intervention and control groups with respect to PA. The quality of the evidence in the included studies was low due to high risk of bias, high heterogeneity (inconsistency), and imprecision (all pooled CIs included 0 and were statistically non-significant).

Müller and Khoo reviewed non-face-to-face PA interventions, such as phone counseling, newsletters, and computer-tailored PA advice letters [57]. The interventions were generally effective; of 16 interventions, 14 reported significant improvements in PA. However, no studies obtained objective measures of PA.

Otmanowski and Chase reviewed primary care-based PA interventions, such as counseling, goal setting, and education [58]. The authors explained that such interventions might be expected to be effective because the participants—older adults—valued and respected their health provider’s advice and visited their physician’s office frequently throughout the year, which created opportunities for the primary care providers to deliver PA interventions. Twenty-one studies were included in the review. A pooled analysis reported a standardized mean difference effect size of 0.27 (95% CI 0.15, 0.39, *p* < 0.01). The authors considered this to be a small to medium effect. Most of the interventions included in this review lasted at least 6 months, and many lasted at least 12 months. Fourteen of the twenty-one studies, however, relied exclusively on self-reported measures of PA. Despite this limitation, the authors concluded that primary care providers could significantly impact the overall health of their older adult patients by providing interventions that increase PA levels.

Sansano-Nadal et al. reviewed exercise-based PA interventions such as resistance training, balance training, and walking [59]. Unlike the other reviews, they limited their analysis to studies with at least six months of post-intervention follow-up. Most of the reviewed studies measured PA using self-reports. The results showed an effect on PA at the six-month follow-up (SMD 0.30; 95% CI 0.15 to 0.44) but not at the one- and two-year follow-ups. The authors found that exercise interventions had small clinical benefits in community-dwelling older adults and that a decline in improvement could be observed after six months of the intervention cessation.

## Discussion

This umbrella review provided an overview of evidence on PA-promoting interventions for community-dwelling older adults, with a light shed on what might be effective in promoting PA among older adults in Saudi Arabia. We summarized the results of 15 systematic reviews covering a multitude of interventions, including eHealth/mHealth (n = 5) and other interventions (n = 10). Several reviews reported that PA- or SB-based interventions were effective in the short term but with wide heterogeneity in findings and methodologies. There were limited studies on documented PA- or SB-based interventions that could be effective for one year or more after the intervention.

Overall, the reviews suggest that some interventions, including mobile apps, automated messaging, and online resources, may modestly increase PA in the short term (e.g., ≤ 3 months) [45–48, 50, 51, 54, 55, 57, 59]. There is also some evidence that interventions carried out in primary care settings, including counselling, goal setting, and education, may increase PA in the long term [58]. The overwhelming majority of studies found in the literature were conducted in Western countries (Table 1). It should not be assumed that the results of the reviewed studies are generalizable to other countries such as Saudi Arabia, which have different geographical, climate, and cultural circumstances. There is evidence of context-specific barriers to higher levels of PA among the Gulf Cooperation Council (GCC) countries—Bahrain, Kuwait, Oman, Qatar, Saudi Arabia, and the United Arab Emirates [83]. These barriers include high temperatures that limit the safety of outdoor exercise during much of the day, high levels of urbanization that promote dependence on motor vehicles rather than active transport, and some cultural barriers [83].

Our study has several potential applications for promoting PA in Saudi Arabia. The healthcare system in Saudi Arabia is undergoing a major transition, aiming to prioritize disease prevention and public health in the new model of care [84]. Given the challenges resulting from increasing rates of chronic diseases that Saudi Arabia and other GCC countries face [85], it will be necessary to make decisions on how to scale up the prevention of noncommunicable diseases even with incomplete or inconclusive evidence.

We recommend a three-pronged approach. First, given the lack of definitive evidence in the literature and the paucity of studies conducted in GCC countries, we suggest promoting low-cost interventions that theory suggests are probably effective and that have at least some evidence base in the global literature. PA- and SB-based interventions delivered to older adults in the primary care setting may be one such intervention. As discussed in the review by Otmanowski and Chase [58], there is some evidence that such interventions may have small to modest effects on increasing PA among community-dwelling older adults. The PA-promoting interventions could be relatively easily implemented in Saudi Arabia, where all citizens have free access to government-funded health services [86]. The Ministry of Health operates a network of primary health clinics that provide public health care services [87]. The Ministry’s policies can be designed to encourage the delivery of PA-based behavioral counseling in primary care, thus potentially increasing PA [88].

Second, we recommend conducting high-quality studies to build an evidence base to facilitate future decision-making. After pilot testing to identify preliminary evidence of effectiveness in short-term studies, the selected multi-component interventions should be tested for effectiveness in older Saudi populations. Such research could use a lengthy follow-up to identify whether any intervention effects are likely to be sustained. Evidence from these high-quality studies is expected to increase investments and efforts to increase PA, reduce SB, and guide evidence-driven health policy. The Saudi Vision 2030 emphasizes the key priorities for scientific research and quality of life programs [31]. The recent national research priorities included health and wellness research as one of the four top national priorities for the next decade. In addition, the use of technologies for health purposes has been extensively expanded in Saudi Arabia during the last five years [89].

Third, we suggest focusing on older adults and other age groups in parallel. PA promotion and SB reduction interventions for older adults cannot be viewed in isolation from those targeting younger age groups. The recent movement practice guidelines for Saudi Arabia recognized the need for the life-time approach to movement behavior promotion and for providing opportunities for all the age groups—from infants to older adults—to engage in the appropriate intensity movement activities [30]. The current interventions that focus on young and working age adults need to support creation of the movement culture that would facilitate implementation of the interventions promoting PA and reducing SB in community-dwelling older adults, as Saudi population ages.

One strength of this umbrella review is the large number of systematic reviews included to synthesize the evidence around interventions that aim to increase PA and reduce SB. To our knowledge, this is the first umbrella review to draw on lessons and implications from the global literature on what might be effective in promoting PA among older adults in Saudi Arabia. However, our study has some limitations that should be acknowledged. Many of the primary studies included in the reviewed sources have methodological shortcomings, such as the absence of a control group, reliance on self-reported PA and SB, failure to blind participants, failure to blind interventionists, failure to blind assessors, and/or failure to fully report on prespecified outcomes. These limitations might impact the evidence generated from these studies. Also, as noted above, the generalizability of these studies is questionable. In addition, we did not consider searching for grey literature and non-English articles. We assumed that the most reliable literature was published in English and indexed in the databases we searched. Future research might consider the grey literature and non-English studies to capture any evidence that might have been excluded from this umbrella review. Finally, we did not assess the quality of the included systematic reviews. Further studies are encouraged to address these limitations to provide solid conclusions.

## Conclusion

In this umbrella review, we assessed the evidence of the effectiveness of interventions aiming to increase PA and/or reduce SB among community-dwelling older adults. There is some evidence that such interventions may be effective in the short term (≤ 3 months), but high-quality evidence on long-term effects is largely lacking. Most reviews were skewed heavily toward studies conducted in Western communities. In the short term, promoting promising and low-cost interventions is reasonable from a public health perspective, even if rigorous evidence of effectiveness is lacking. High-quality studies are needed to assess the validity of our findings among the Saudi population to identify cost-effective strategies to increase PA and reduce SB.

## Data Availability

All data generated or analyzed during this study are included in this published article.

## Abbreviations

CI: Confidence interval;
COPD: Chronic obstructive pulmonary disease;
GCC: Gulf Cooperation Council;
PA: Physical activity;
SB: Sedentary behavior
